# Association of *APOC1* with cortical atrophy during conversion to Alzheimer’s disease

**DOI:** 10.1007/s11357-025-01695-6

**Published:** 2025-05-15

**Authors:** Sewook Oh, Sunghun Kim, Jun Pyo Kim, Sang Won Seo, Bo-yong Park, Hyunjin Park

**Affiliations:** 1 Department of Electrical and Computer Engineering, Sungkyunkwan University, Jangan-gu, Suwon, Gyeonggi-do 16419 Republic of Korea; 2 Center for Neuroscience Imaging Research, Institute for Basic Science, Jangan-gu, Suwon, Gyeonggi-do 16419 Republic of Korea; 3 Department of Brain and Cognitive Engineering, Korea University, Seongbuk-gu, Seoul, 02841 Republic of Korea; 4 BK21 Four Institute of Precision Public Health, Seoul, Republic of Korea; 5 Alzheimer’s Disease Convergence Research Center, Samsung Medical Center, Gangnam-gu, Seoul, 06351 Republic of Korea; 6 Department of Neurology, Samsung Medical Center, Sungkyunkwan University School of Medicine, Gangnam-gu, Seoul, 06351 Republic of Korea; 7 Neuroscience Center, Samsung Medical Center, Gangnam-gu, Seoul, 06351 Republic of Korea

**Keywords:** Alzheimer’s disease, Conversion, *APOC1*, Gene expression, Cortical thickness

## Abstract

Alzheimer’s disease (AD) is a prevalent neurodegenerative disorder, with its progression influenced by aberrant gene expression and alterations in the brain network topology. Although *APOE* has been extensively studied in relation to AD, the role of *APOC1* remains relatively underexplored. This study investigated the impact of *APOC1* on changes in cortical thickness (CTh) during conversion to AD in a longitudinal setting. Using a normative modeling approach, we examined changes in CTh in patients with mild cognitive impairment (MCI). The spatial patterns of CTh changes were then correlated with *APOC1* mRNA expression levels. We estimated the time to conversion to AD and compared progression rates between the low and high *APOC1* expression groups. Finally, mediation analysis was performed to assess the indirect effects of *APOC1* expression on memory function via CTh changes. In patients with MCI and AD, reduced CTh was observed in the limbic and default mode regions, with a notable impact on the entorhinal cortex, parahippocampus, and fusiform gyrus when comparing baseline and follow-up measurements. The degree of change in CTh was significantly associated with *APOC1* expression, with the paralimbic regions identified as particularly vulnerable. Furthermore, the high *APOC1* expression group demonstrated more rapid conversion to AD than that observed in the low expression group. Mediation analysis indicated a trend suggesting that *APOC1* expression indirectly affected memory and cognitive function through its influence on CTh. These results highlight the potential of *APOC1* as an additional focus of AD research, offering insights into the genetic influences on AD pathology.

**Supplementary Information:**

The online version contains supplementary material available at 10.1007/s11357-025-01695-6.

## Introduction

Alzheimer’s disease (AD) is a genetically heterogeneous condition with high heritability that involves complex interactions between multiple genes [1]. Among the genetic factors, *APOE* ε4 is a well-established factor central to AD progression and risk prediction [2, 3]. Numerous genome-wide association studies (GWAS) and meta-analyses have demonstrated a strong correlation between AD risk and *APOE* ε4 [4, 5].

Recent studies have broadened their focus beyond *APOE* ε4 to investigate additional genes in linkage disequilibrium (LD) by identifying additional genetic risk factors for AD [6, 7], including the apolipoprotein family [8–10]. In this family, apolipoprotein C1 (*APOC1*) regulates lipid levels and cognitive functions [11]. The proximity of *APOC1* to *APOE* on chromosome 19 raises the possibility that observed associations between *APOC1* and AD may primarily reflect LD; however, emerging evidence suggests that *APOC1* may exert independent effects on AD pathogenesis [12]. Multiple studies have confirmed that *APOC1* is involved in amyloid-β (Aβ) accumulation and contributes to cognitive decline and memory impairment, similar to the effects observed with *APOE* ε4 [13, 14]. Furthermore, *APOC1* insertion allele (H2) is significantly associated with an increased risk of late-onset AD, especially when interacting with *APOE* ε4, indicating that *APOC1* could be considered as a target for AD pathology [15].

Brain atrophy is a validated marker for identifying the risk of developing AD [16–19], assisting clinicians in making decisions regarding diagnosis and prognosis [20]. Specifically, cortical thickness (CTh) is key to assessing brain atrophy in AD, with age-related decreases closely linked to cognitive and memory impairments [18] as well as AD conversion [21, 22]. Memory decline in patients with AD is specifically associated with atrophy in the temporal lobe, whereas abnormal executive control is related to widespread cortical atrophy, with pronounced involvement of the parietal and temporal lobes [23].

This evidence collectively suggests the necessity for imaging-genetics studies that integrate imaging and genetic phenotypes to investigate the neurological mechanisms of AD. Previous studies identified specific genetic variants associated with regional brain atrophy and cognitive decline [24]. In the *TOMM40*-*APOC1*, single-nucleotide polymorphisms (SNPs), including rs4420638, rs56131196, and rs157582, have been identified as significant loci associated with hippocampal volume (HV) atrophy and accelerated cognitive decline in patients with mild cognitive impairment (MCI) [24]. Moreover, *APOC1* polymorphism influences HV, which may have a significant effect on brain atrophy compared to that of *APOE* [25]. This suggests that *APOC1* and *APOE* may have differential effects on brains that are vulnerable to AD progression. However, the relationship between *APOC1* expression and brain atrophy lacks evidence and requires further investigation.

To address these gaps, we conducted a comprehensive study to consolidate the knowledge, imaging, and phenotypes of AD. We examined the association between *APOC1* expression and CTh, focusing on its impact on cognitive function and memory. We analyzed the spatial correlation between *APOC1* expression and cortical atrophy and probed the potential link between *APOC1* levels and accelerated conversion to AD and cognitive decline. Vertex-level CTh measurements were analyzed using generalized additive models for location, scale, and shape (GAMLSS) normative modeling. This statistical approach was employed to assess conversion (i.e., change from MCI to AD) risk associated with *APOC1* expression. Our study elucidated the role of *APOC1* in the neurodegenerative processes underlying AD, potentially confirming its use as a biomarker for early intervention in a multimodal setup spanning the gene-imaging-phenotype spectrum.

## Materials and methods

### Study participants

Our study included individuals with MCI and analyzed baseline and follow-up data to explore the genetic risk profiles and pathways through which *APOC1* may contribute to AD conversion. Imaging and phenotypic data were obtained from the Alzheimer’s Disease Neuroimaging Initiative (ADNI) Database (https://adni.loni.usc.edu/) [26]. We included 170 individuals at risk of AD conversion, with imaging and phenotypic data obtained at both baseline and follow-up. Inclusion criteria were a follow-up period of at least 1 year, no cognitive improvement at follow-up (i.e., increased Mini-Mental State Examination [MMSE] score at follow-up), and inclusion of SNP measurements. Cases of cognitive reversion (i.e., MCI to cognitively normal [CN]) were excluded to consider only individuals at risk of conversion to AD. Follow-up sessions were selected as either the first AD diagnosis in uncensored cases or the last MCI session in censored cases. Among the 170 individuals, blood gene expression data for *APOC1* were available for 95. Additionally, a reference group of 636 individuals with CN status was included. The demographic information of the study participants is presented in Table 1.

**Table 1:** Demographic and clinical information of the study participants

	Cognitively impaired		Cognitively normal (CN)	*P*-value (baseline vs. CN)
	Baseline	Follow-up		
*n*	170	170	636	-
Age (years)	73.7 ± 7.35	76.3 ± 7.46	75.3 ± 6.80	0.007
Sex (male/female)	94:76	94:76	270:366	0.003
MMSE	27.5 ± 1.93	25.3 ± 3.37	29.0 ± 1.28	< 0.001
CDRSB	1.57 ± 0.90	3.57 ± 1.74	0.08 ± 0.22	< 0.001
ADNI-MEM	0.05 ± 0.62	−0.38 ± 0.86	1.14 ± 0.67	< 0.001
*APOE* ε4 carrier (carrier/non-carrier)	94:76	94:76	196:440	< 0.001
Education (years)	15.8 ± 2.64	15.8 ± 2.64	16.7 ± 2.46	< 0.001
Follow-up time (years)		2.62 ± 1.44		
Conversion (%)		111 (65.3%)		

*ADNI* Alzheimer’s disease neuroimaging initiative, *MMSE* mini-mental state examination, *CDRSB* clinical dementia rating scale sum of boxes, *MCI* mild cognitive impairment, *ADNI-MEM* Alzheimer’s disease neuroimaging initiative composite memory score

### Genetic data acquisition

#### Genotype

Genotypes were obtained from genomic DNA samples extracted from the peripheral blood of ADNI participants. For ADNI-1 samples, Illumina chips were utilized for genotyping, and intensity data were processed using the GenomeStudio software (Illumina). Additionally, ADNI-GO/2 samples were genotyped and processed using the Illumina Human OmniExpress BeadChip and GenomeStudio v2009.1. Furthermore, ADNI-3 samples were genotyped and processed using the Illumina Infinium Global Screening Array v2 (GSA2) and GenomeStudio v2.0.4 (Illumina) [27].

#### ADNI blood gene expression

*APOC1* mRNA expression was profiled from the peripheral blood samples of ADNI participants. The Affymetrix Human Genome U219 Array (www.affymetrix.com) was employed for the expression profiling. Peripheral blood samples were collected in PAX gene tubes for RNA analysis. After quality control, the Affymetrix HG U219 Array contained 530,467 probes corresponding to 49,293 transcripts. All the probe sets were mapped and annotated using the hg19 genome build [28]. We used only the gene expression data measured from the MCI baseline session, which was matched with the corresponding imaging session.

#### Allen brain gene expression

The Allen Human Brain Atlas (AHBA) comprises complete normalized microarray datasets of six brains [29]. After quality control and normalization, the gene expression profile contained 62,000 probes, with 93% of known genes represented by at least two probes. Each gene expression value corresponded to an anatomical region mapped to the standard Montreal Neurological Institute coordinates. The AHBA data were used to extract transcriptional profiles across brain regions, providing a database of microarray-based log expression levels for 15,632 genes from the *post-mortem* brains of six donors without psychiatric or neuropathological disorders.

### Genetic data processing

#### Genotype quality control and imputation

The ENIGMA protocol (https://enigma.ini.usc.edu/) was utilized to perform the imputation and quality control processes on the genotype data. First, we eliminated any strands of ambiguous SNPs and screened for low minor allele frequency (< 0.01), genotype call rate (< 95%), and Hardy–Weinberg equilibrium (< 1e−6). The Michigan imputation server [30] was used to impute the genotype using 1000 Genomes Phase 3 v5 [31] as the reference panel, and phasing was performed using the Eagle v2.3 [32]. Additionally, PLINK 1.9 [33] was used for quality control. We employed the ANNOtate VARiation (ANNOVAR) to annotate our target SNPs [34] and SNPNexus to evaluate the structural and functional roles of the SNPs [35].

#### Gene expression processing and analysis

Gene expression data from ADNI (i.e., *APOC1* in peripheral blood) were log2-scaled and adjusted for biological and technical covariates (i.e., age, sex, RNA integrity value, and batch) to mitigate the potential influence of confounding factors on the relationship between *APOC1* expression and CTh changes. Confounding effects of *APOE* were modeled by including *APOE* gene expression and *APOE* ε4 status as additional covariates. We applied a linear model to the gene expression data with covariates, calculated the model residuals, and added them back to the mean gene expression, resulting in adjusted gene expression values. For gene expression measured with multiple probes in *APOC1*, low-hit rate probes were excluded, and the expression values were averaged and adjusted across the remaining probes. For *APOE* gene expression, we used biological and technical covariates and *APOE* ε4 status as covariates in the adjustment regression.

### Image acquisition

MRI data from ADNI were scanned using Philips Medical Systems, Siemens Medical Solutions, and GE Healthcare 3 T scanners. T1-weighted (T1w) images were acquired with a three-dimensional (3D) magnetization-prepared rapid acquisition gradient echo sequence for Philips and Siemens medical systems scanners (Philips: repetition time [TR] = 6.8 ms, echo time [TE] = 3.2 ms, and flip angle = 9°; Siemens: TR = 2300 ms; TE = 2.98 ms, flip angle = 9°). For the GE Healthcare scanners, T1w images were collected using a 3D accelerated sagittal inversion recovery-prepared fast spoiled gradient recalled sequence (TR = not provided, TE = min full, flip angle = 11°).

### Image preprocessing

T1w scans were preprocessed using micapipe version 0.2.3, which is an open-source processing pipeline for multimodal MRI data [36]. After intensity nonuniformity correction and normalization [37], the scans were skull-stripped. Additionally, tissue types, including gray matter, white matter, and cerebrospinal fluid, were segmented. The cortical surface was generated from native T1w scans using FreeSurfer version 7.3.2 [38]. CTh was then estimated from the generated cortical surfaces by measuring the distance between the white and pial surfaces [39]. The CTh values were transformed to the fsaverage5 surface template (20,484 vertices), and site effects were adjusted using ComBat harmonization [40].

### Normative modeling and centile score estimation

Normative modeling of age-related CTh differences was performed using data from the CN group employing the GAMLSS [41, 42]. The GAMLSS is useful for modeling complex and nonlinear patterns [43–46]. It is also robust in approximating population-level developmental trajectories and enables providing a fine-grained, individualized assessment of features. In this study, we employed this approach to quantify degenerative changes in CTh by constructing vertex-wise normative trajectories from 636 CN participants. Following the recommendations for the normative modeling of neuroimaging data [47], we applied a four-parameter sinh-arcsinh (SHASH) distribution [48]. Vertex-wise normative modeling of CTh based on age, the interaction of sex and age, and intracranial volume (ICV) was performed as follows:

1 $$\begin{array}{c}\text{CTh}\sim\text{SHASH}\left(\mu,\sigma,\nu,\tau\right),\\\mu=\beta_\mu+\beta_{\mu,\text{age}}f\left(\text{age}\right)+\beta_{\mu,\text{age}\ast\text{sex}}f\left(\text{age}\ast\text{sex}\right)+\beta_{\mu,\text{ICV}}ICV,\\\text{log}\left(\sigma\right)=\beta_\sigma+\beta_{\sigma,\text{age}}f\left(\text{age}\right)+\beta_{\sigma,\text{ICV}}ICV,\text{log}\left(\nu\right)=\beta_\nu,\text{log}(\tau)=\beta_\tau\end{array}$$

where $f$ is a non-linear function (i.e., P-spline), $\beta$ denotes the regression coefficient, and $\mu$, $\sigma$, $\nu$, and $\tau$ indicate the multi-parameters that control location, scale, skewness, and kurtosis respectively. The GAMLSS model fitting was implemented using the *gamlss* R package [41]. To quantify the individual deviations of patients from the normative trajectory, we calculated centile scores for CTh across various age groups. The individual deviations of patients from the normative trajectory were evaluated using centile scores of CTh that were derived by comparing each individual’s CTh to the normative distribution, determining their positions within it. A low score reflected a thinner CTh, whereas a high score indicated a thicker CTh relative to the reference group (i.e., CN).

### CTh changes in patients at risk of conversion to AD

We employed a surface-based linear mixed-effects model [49] for each vertex to evaluate the effect of time changes on CTh centile scores as follows:

2 $${\text{CTh centile score}}_{ij} = {\beta }_{0}+{\beta }_{1}{\text{Time}}_{ij}+{z}_{i}+{\varepsilon }_{ij}$$

where ${\beta }_{0}$ represents the intercept, ${\beta }_{1}$ is the fixed effect of time (i.e., baseline to follow-up), ${z}_{i}$ denotes the random intercept for individual $i$ to account for inter-individual variability, $j$ denotes each vertex, and ${\varepsilon }_{ij}$ is the error term. A beta map (i.e., CTh change map) was generated through ${\beta }_{1}$ for each vertex, and their effects were stratified using seven functional networks, including visual, somatomotor, dorsal attention, ventral attention, limbic, frontoparietal, and default mode networks [50]. This approach allowed for flexible modeling with varying follow-up intervals. Significant vertices were identified using the random field theory. Surface-based findings were corrected for multiple comparisons to control for family-wise error rate (i.e., *p*_FWE_ < 0.05), with a cluster threshold of *p* = 0.001. With the significantly identified vertices, we averaged the CTh percentile scores for further analysis.

### Reliability of CTh change map in mapping cognitive correlates and AD conversion

To explore cognitive processes associated with the observed CTh change map, we conducted cognitive decoding using NeuroSynth [49, 51]. The CTh change map was decoded to identify the cognitive terms that strongly correlated with the brain regions exhibiting changes in CTh. After excluding anatomical and methodological terms and merging duplicate words, the top 100 remaining terms were ranked by their correlation strength. These terms were then visualized as word clouds to provide an overview of the cognitive profiles associated with the CTh changes. To further assess the impact of CTh on conversion risk, the Cox proportional hazards (CPH) model was applied, incorporating the baseline average CTh centile score along with covariates such as age, sex, MMSE, and education level.

### APOC1 expressions associated with CTh changes

We performed a transcriptomic association analysis to assess whether CTh changes in patients were related to the expression of a specific gene (*APOC1*). We hypothesized that a thin CTh would be associated with a high regional *APOC1* mRNA expression. We obtained cortical *APOC1* mRNA expression data from the AHBA [29] using the Abagen toolbox [52]. We utilized the mean expression of *APOC1* across the six donors, and the expression data were mapped to the Schaefer atlas with 300 parcels [53], where missing values were imputed using the median value. As only left hemisphere expression was available for four of the six donors, we used only the left hemisphere, as per the previous studies [54, 55]. Mean expression data across donors were mapped to the fsaverage5 surface and correlated with the CTh change map. The association between *APOC1* expression and CTh changes was assessed using Pearson’s correlation, and the statistical significance of the correlations was evaluated using 1000 nonparametric spin permutation tests [56, 57].

### Gene ontology analysis

To investigate the co-varying neuromolecular properties of CTh changes, we conducted a gene ontology (GO) analysis. We correlated the CTh change map with *post-mortem* gene expression maps from the AHBA. We selected 100 genes with the highest negative correlations, prioritizing those associated with cortical thinning and its related symptoms. These genes were then subjected to a GO analysis (https://geneontology.org/) [58] to assess their biological processes, molecular functions, and cellular components [59, 60]. We also compared these genes using cell-type-specific expression analysis to identify candidate cell populations associated with the input gene lists, assessing significance with a *z*-score modification of Fisher’s exact test and applying false discovery rate (FDR) correction (http://doughertytools.wustl.edu/CSEAtool.html) [61].

### Estimation of time to conversion

Time to conversion is a critical measure for identifying the progression from MCI to AD, and the accurate prediction of this timing is essential for subsequent analyses. Time to conversion was defined as the duration from baseline to the occurrence of the conversion event. Given that conversion times are interval-censored (i.e., occurring between the last MCI diagnosis and the first AD diagnosis), we estimated this time based on imputed survival times, following the methodology of a previous study [62]. Unlike simple midpoint imputation, the multiple imputation strategy first estimates the underlying survival function and then stochastically imputes conversion times for subjects based on their observed intervals [63, 64]. This method not only accounts for the uncertainty in exact conversion times but also preserves the distributional characteristics of the data, making it a more reliable strategy for AD conversion studies compared to naive imputation techniques. A Weibull accelerated failure time model was fitted using clinical covariates (i.e., age, sex, MMSE, *APOE* ε4 status, and education level) using the *icenReg* R package [65], thereby converting interval-censored data into right-censored data suitable for conventional survival analysis.

### Association between *APOC1*, SNPs, and AD conversion

To examine whether *APOC1* expression levels influence the rate of conversion, we conducted a Kaplan–Meier analysis using peripheral blood *APOC1*, controlling for the effects of *APOE* and *APOE* ε4 status. *APOC1* expression levels in peripheral blood, obtained from the ADNI dataset, were used to categorize participants into low- and high-expression groups based on the median value. SNPs located within the *APOC1* locus were obtained from the ADNI dataset using hg19 coordinates. These SNPs were converted to hg38 coordinates using CrossMap [66], and those located within ± 1 kb of the *APOC1* locus were included in the analysis. To assess whether the *APOC1* SNPs influencing conversion are independent of *APOE* ε4, we conducted an LD analysis using Haploview [67]. Participants were categorized into non-variant and variant groups, and their conversion trajectories were compared using a log-rank test. The FDR was employed to adjust for multiple comparisons. We additionally examined the interaction between *APOC1* expression and *APOE* ε4 status by analyzing time to conversion using Kaplan–Meier survival analysis with four groups stratified based on *APOC1* expression levels (high or low) and the *APOE* ε4 status (positive or negative).

### Mediation analysis

We performed mediation analyses to assess whether elevated *APOC1* expression levels in the peripheral blood of patients with ADNI mediate accelerated cognitive decline via CTh. We also controlled for the effects of *APOE* and *APOE* ε4 status. Unlike the AHBA dataset, the ADNI database provided gene expression data from peripheral blood for each participant, which allowed us to perform a mediation analysis. To this end, we calculated the annual changes in memory performance and symptom severity. The memory and cognitive functions were evaluated using the ADNI composite MEMory score (ADNI-MEM) and the clinical dementia rating scale sum of boxes (CDRSB). The CDRSB is a widely used tool for assessing dementia severity across multiple domains [68], and the ADNI-MEM is a validated composite score of episodic memory developed by the ADNI core to summarize outcomes from multiple memory assessments [69]. ADNI-MEM is an effective measure for tracking cognitive changes and predicting MCI-to-AD conversion and is associated with memory-related neuroimaging parameters [69]. The significance and robustness of the findings were assessed using the bootstrapping analysis with 5000 iterations, conducted with the *lavaan* package in R, with all regression weights standardized [70].

## Results

### Change in CTh during AD conversion

**Fig. 1:**
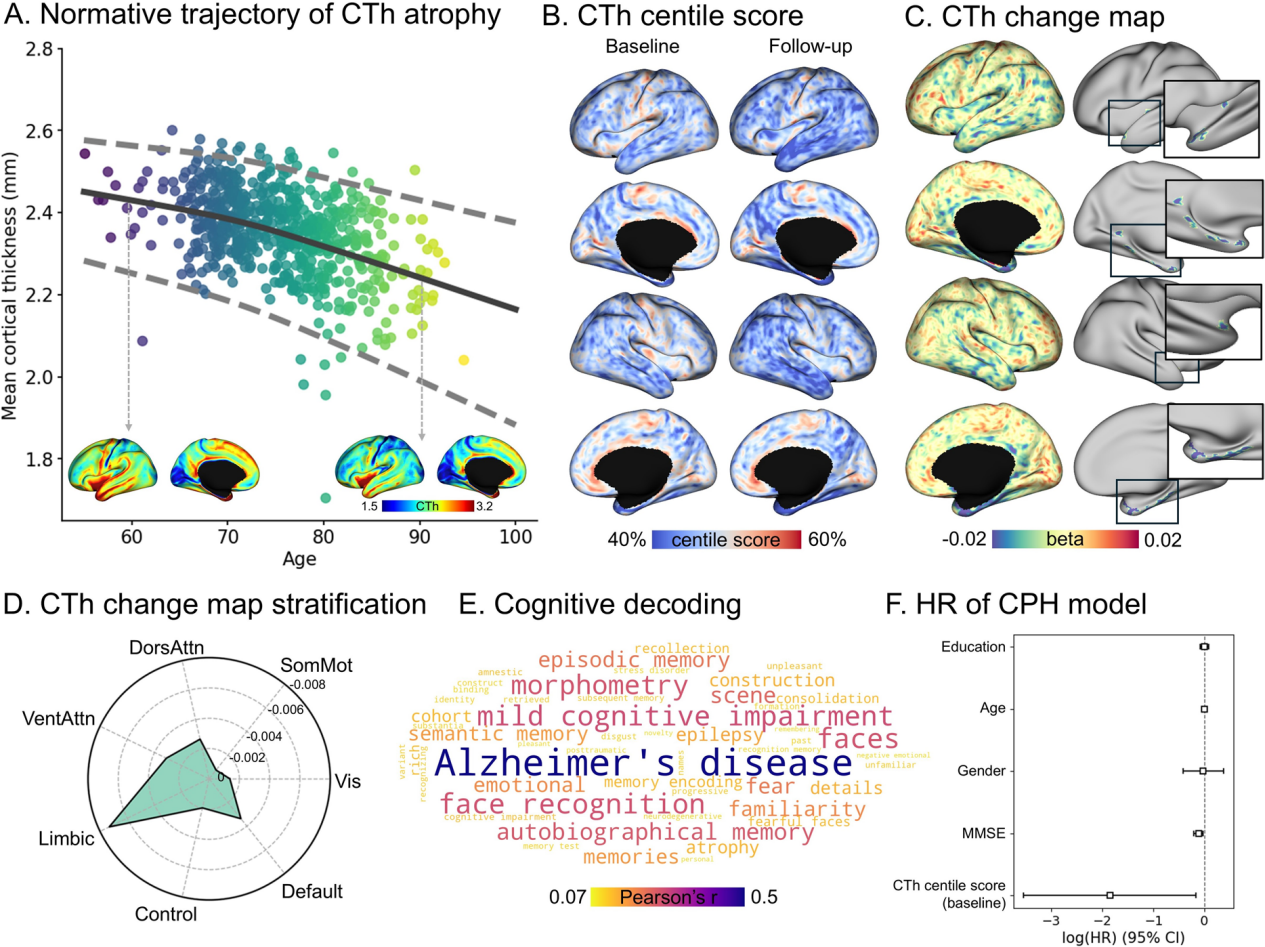
CTh changes in patients at risk of conversion to AD. **A** Mean CTh changes across ages in 636 individuals with CN status. Representative CTh patterns are displayed on brain surfaces. The colored dot indicates the age of the participants with CN status. **B** CTh centile scores of individuals with cognitive impairment (baseline and follow-up). Colors indicate the centile score of MCI at baseline (left) and follow-up (right). **C** The CTh change map of individuals with cognitive impairment. **D** The degree of CTh change stratified according to functional communities. **E** Cognitive terms related to the CTh change map. **F** HR of CTh and covariates from CPH model. CTh, cortical thickness; AD, Alzheimer’s disease; CN, cognitively normal; Vis, visual; SomMot, somatomotor; DorsAttn, dorsal attention; VentAttn, ventral attention; HR, hazard ratio; CPH, Cox proportional hazards; MMSE, mini-mental state examination; CI, confidence interval

We evaluated cortical atrophy in the normal-aging group by generating vertex-wise normative trajectories using the GAMLSS model in 636 participants with CN status. The mean CTh across all vertices demonstrated a nonlinearly decreasing trajectory with age (Fig. 1A). By leveraging normative models, we quantified the level of CTh atrophy in patients at each vertex using the centile scores. We identified that individuals with cognitive impairment exhibited greater overall atrophy (extensive blue regions) than normative trajectories, with the extent of atrophy aggravating at follow-up (Fig. 1B). We also computed the CTh percentile scores for the stable MCI and conversion (i.e., MCI to AD) subgroups, and the conversion subgroup exhibited greater cortical thinning than those demonstrated by the stable MCI subgroup (Fig. S1). To quantify the CTh changes during conversion, we derived a CTh change map (i.e., beta-map) using a linear mixed effect model (Fig. 1C, left) and identified 194 significant vertices (*p*_FWE_ < 0.05, cluster *p* = 0.001; Fig. 1C, right). The medial and lateral temporal regions, notably the entorhinal cortex, parahippocampus, and fusiform gyrus, exhibited significant reductions in CTh. When we stratified the CTh change map using seven intrinsic functional communities, we observed the most pronounced atrophy in the limbic region, followed by the default mode and attention networks (Fig. 1D). Decoding the cognitive correlates of the CTh change map further corroborates the strong association with terms like “AD,” “MCI,” “morphometry,” and “memory,” confirming the relevance of these CTh change patterns to AD conversion (Fig. 1E). Finally, to assess whether the CTh percentile scores of the identified vertices could serve as markers of conversion risk, we fitted the CPH model to known risk factors. The results indicated that our CTh feature was statistically significant while controlling for other risk factors, with a log hazard ratio (HR) of −1.85 (95% confidence interval = [−3.54, −0.16], *p* = 0.03; Fig. 1F).

### *APOC1* expressions are spatially associated with CTh thinning

**Fig. 2:**
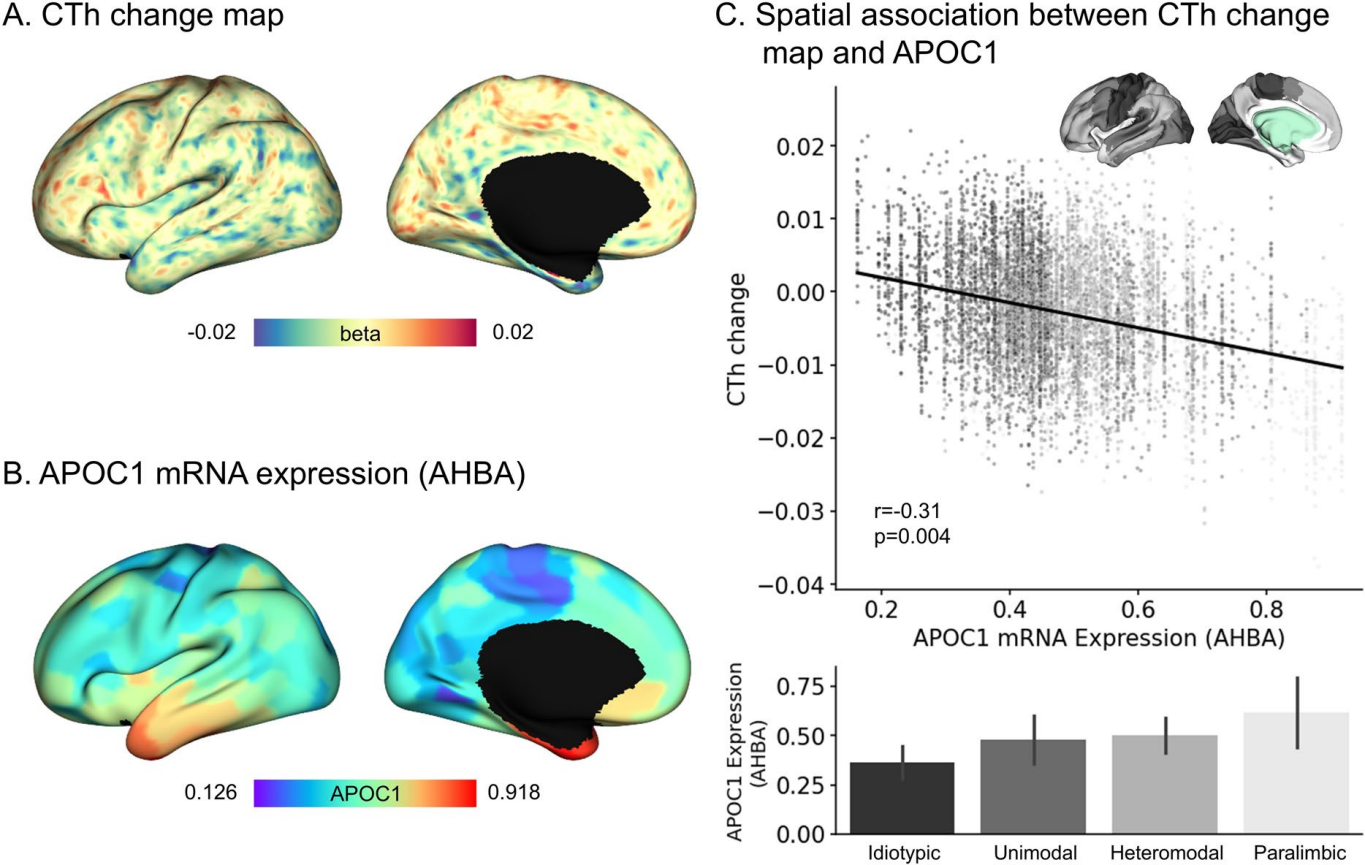
The association between CTh change and *APOC1* expression. **A** CTh change map and (**B**) *APOC1* mRNA expression extracted from AHBA displayed on brain surfaces. **C** A spatial correlation between the CTh change map and *APOC1* expression illustrated with a scatter plot. The color is coded according to prior models of cortical hierarchy. The bar plot indicates the *APOC1* expression according to cortical hierarchies. CTh, cortical thickness; AHBA, Allen human brain atlas

To examine the regional effects of *APOC1* expression on CTh changes, we used whole-brain gene expression data for *APOC1* from the AHBA. We associated the spatial patterns between the CTh change map and *APOC1* expression within the cortex (Fig. 2A–B) and observed a significant correlation (*r* = −0.31, *p*_spin_ = 0.004) with regions of high *APOC1* expression displaying thin CTh (Fig. 2C). Interestingly, *APOC1* expression decreased along the cortical hierarchy [71] (Fig. 2C), suggesting that high-order paralimbic areas are significantly vulnerable to the disease.

### Results of ontology analysis

**Fig. 3:**
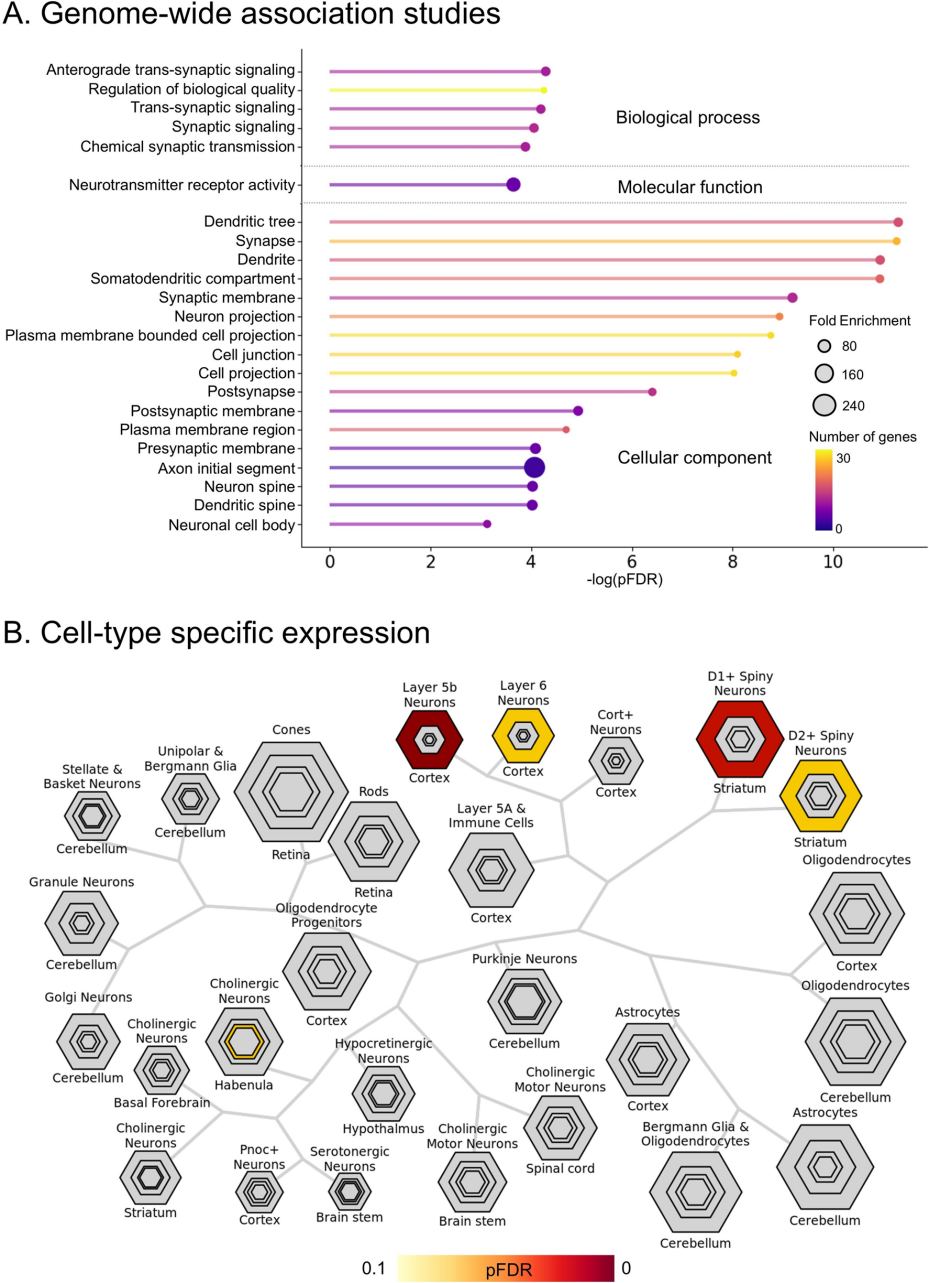
Gene ontology analysis. **A** Genes correlated with the CTh change map are further associated with gene expression profiles from genome-wide association studies. The *x*-axis displays the negative log-transformed FDR-corrected *p*-values. Circle size represents the fold enrichment. The color bar indicates the number of genes contained within each term. **B** Cell-type-specific expression analysis identified candidate cell populations linked to genes correlated with the CTh change map. Hexagon size demonstrates the proportion of genes expressed in each tissue, with outer to center hexagons representing increasing enrichment stringency (specificity index threshold [pSI], 0.05, 0.01, 0.001, 0.0001). The color bar indicates the FDR-corrected *p*-value. CTh, cortical thickness; FDR, false discovery rate

GO analysis revealed possible neurobiological connections in our macroscale findings (i.e., CTh change map). Among the identified genes related to the CTh change map, *APOC1* demonstrated the third-strongest correlation (Table S1). The 100 genes that exhibited the highest correlation with CTh changes exhibited pathways involving synaptic signaling under chemical synaptic transmission, emerging as the predominant processes (*p*_FDR_ < 0.05; Fig. 3A), consistent with the findings of a previous study [72]. The enrichment of genes associated with synaptic transmission, neuronal structure, and neurotransmitter receptor activity suggests that disruptions in synaptic integrity and neuronal connectivity underlie the structural alterations observed in the CTh change map. Furthermore, cell type-specific expression analysis indicated that these genes were enriched in cortical cells and the striatum (Fig. 3B). Specifically, these genes were enriched in D1 and D2 spiny neurons in the striatum as well as in neurons in cortical layers 5b and 6 (*p*_FDR_ < 0.05). The findings underscore the potential role of the related genes that may contribute to explaining neurobiological processes on cortical atrophy.

### SNPs in *APOC1* and their association with rapid conversion to AD

**Fig. 4:**
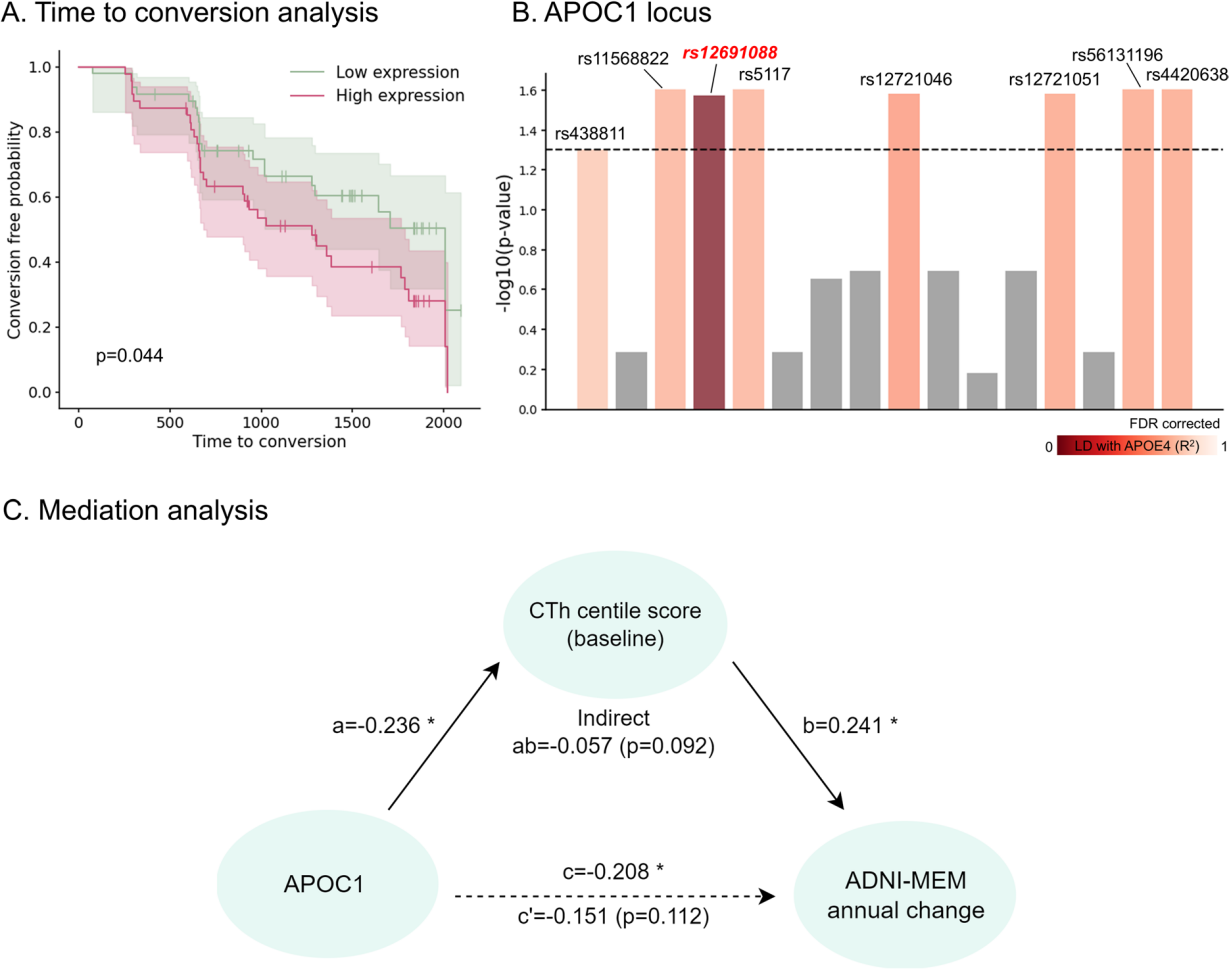
*APOC1* related to AD conversion and cognitive function via mediation. **A** Kaplan–Meier plot clearly distinguished low and high *APOC1* expression groups. **B** Analysis of significant SNP in *APOC1* locus (± 1 kb) for conversion risk based on variant status. The color indicates the strength of LD with *APOE* ε4. **C** Mediation analysis assessed the direct and indirect effects of the *APOC1* expression on the annual change of ADNI-MEM mediated by the CTh centile score. Reported values are regression weights, with significance indicated in asterisks. *p-value < 0.05; *AD*, Alzheimer’s disease; *SNP*, single-nucleotide polymorphism; *LD*, linkage disequilibrium; *ADNI-MEM*, Alzheimer’s disease neuroimaging initiative composite memory score; *CTh*, cortical thickness

We further examined whether adjusted *APOC1* expression levels in peripheral blood could serve as a predictor of AD conversion. Kaplan–Meier analysis revealed that the low- and high-expression groups had significantly different conversion trajectories (log-rank test, *p* = 0.044; HR = 1.77; Fig. 4A), with faster conversion in the high-expression group than in the low-expression group. However, no significant differences in baseline CTh centile scores were observed between the groups when multiple corrections were applied (Fig. S2). When *APOE* ε4 status was additionally considered, individuals with high *APOC1* expression showed a significantly faster conversion trajectory compared to low *APOC1* expression individuals for positive *APOE* ε4 status only (*p* = 0.02; Fig. S3). However, there were no significant differences between the high and low *APOC1* expression groups for individuals with negative *APOE* ε4 (*p* = 0.714; Fig. S3). The findings indicate that *APOC1* may further accelerate disease progression in the presence of *APOE* ε4, suggesting a possible interaction between *APOC1* and *APOE* ε4 on AD conversion.

When we categorized the SNPs in the *APOC1* locus into non-variant and variant groups and compared their conversion trajectories using a log-rank test, we discovered that several SNPs exceeded the significance threshold (*p*_FDR_ < 0.05): rs438811, rs11568822, rs12691088, rs5117, rs12721046, rs12721051, rs56131196, and rs4420638 (Fig. 4B). These SNP variants were associated with a rapid conversion outcome (mean HR = 1.97 ± 0.64; Table S2). Given that numerous SNPs located within the *APOC1* locus exhibit high LD with *APOE* ε4, we conducted an LD analysis to examine the relationship between SNPs influencing rapid conversion and the *APOE* ε4 haplotype (Fig. S4). Among these, only rs12691088 was found to be independent of *APOE* ε4 (${r}^{2}$ = 0.024).

### Cortical atrophy mediates the effects of *APOC1* on cognitive impairment

Mediation analysis was conducted to test whether adjusted *APOC1* levels in peripheral blood measured at baseline influenced cognitive impairment through their effects on CTh. We identified a marginal indirect effect (*p* < 0.1) of baseline CTh percentile scores on annual changes in ADNI-MEM (Fig. 4C). Similar results were observed when the same analysis was performed using the CDRSB (Fig. S5). These results suggest that baseline *APOC1* levels in the peripheral blood may modestly contribute to the accelerated deterioration of cognitive and severity scores over time through cortical atrophy.

### Investigation of the mediating role of *APOC1* on hippocampal volume change

We conducted a replication study to determine whether the direct and indirect effects identified in the mediation analysis extend beyond CTh to another key atrophy marker, HV. We assessed hippocampal atrophy in the normal-aging group using the GAMLSS model in 636 participants with CN status. Both left and right HV showed a decreasing trajectory with age (Fig. S6). The *APOC1* mediation results revealed that the left HV produced outcomes for ADNI-MEM (*p* = 0.127) and CDRSB (*p* = 0.114) that were similar to those obtained using CTh, whereas the right HV did not yield significant effects (Fig. S7).

### Control analyses

To determine whether the effects of *APOC1* were distinct from those of *APOE*, we evaluated the influence of *APOE* mRNA expression on the spatial pattern of CTh changes. Moreover, we also investigated whether *APOE* mRNA expression in the peripheral blood affected cognitive and severity scores through cortical atrophy. We discovered that the spatial correlation between the CTh change map and *APOE* mRNA expression from AHBA was significant, but weaker than that of *APOC1* (*APOE*: *r* = −0.20, *p*_spin_ = 0.046, *APOC1*: *r* = −0.31, *p*_spin_ = 0.004; Fig. S8). Moreover, in the mediation analysis, *APOE* expression in the peripheral blood did not demonstrate a correlation with baseline CTh (*p* = 0.78), and an indirect effect on ADNI-MEM or CDRSB was not observed (Fig. S9). Similarly, *APOE* in the peripheral blood did not show any significant mediation configuration in the HV replication study (Fig. S10).

## Discussion

AD progression is influenced by complex genetic interactions, necessitating the investigation of genetic risk factors. In this study, we systematically examined the association between *APOC1* expression and CTh atrophy and their combined effects on the conversion from MCI to AD. We observed reduced CTh levels in the temporal lobe, particularly in the entorhinal cortex, parahippocampus, and fusiform gyrus. Furthermore, significant associations were also noted between these changes and *APOC1* expression in the brain. Elevated *APOC1* levels were particularly notable in the regions affected by CTh atrophy. Additionally, *APOC1* expression in the peripheral blood and specific SNPs within the *APOC1* locus significantly predicted conversion risk, reinforcing its role as a risk factor for AD progression. Ontology analysis revealed that these genetic pathways aligned with the CTh atrophy map, suggesting disruptions in synaptic signaling and neuronal connectivity. Finally, the impact of *APOC1* expression on cognitive decline was mediated by cortical atrophy. Notably, comparisons with *APOE* underscored the distinct role of *APOC1*, underscoring its potential as a biomarker for accelerating disease progression.

Normative CTh trajectories established using the GAMLSS model in healthy aging individuals have helped identify deviations in cortical atrophy in those at risk of conversion to AD. Atrophy in the entorhinal cortex intensifies with disease progression and is correlated with cognitive dysfunction [73], complementing our results. The clinical relevance of these atrophy patterns, evaluated using baseline CTh percentile scoring within the CPH model, confirmed that these patterns are significant predictors of conversion risk and serve as sensitive biomarkers for assessing individuals at an elevated risk of conversion to AD.

GO analyses have elucidated the potential neurobiological mechanisms underlying structural brain changes during AD conversion. In particular, genes associated with changes in CTh were found to be enriched in pathways related to chemical synaptic transmission and neurotransmitter receptor activity. A previous study revealed that neurodegeneration-related alterations in CTh were associated with genes upregulating these pathways, underscoring the critical role of synaptic regulation in AD progression [72]. Additionally, gene enrichment in the molecular function and cellular component categories further highlighted the importance of synaptic transmission, with cell-type-specific analyses indicating that these gene sets are predominantly expressed in cortical and striatal spiny neurons. Other previous transcriptomic studies and investigations into cortical synaptic density in AD have underscored the link between synaptic transmission and AD pathogenesis, demonstrating that disruptions in synaptic integrity contribute to cortical thinning in AD [74, 75]. Our findings, consistent with previous studies [72, 74–76], reinforce the connection between synaptic dysfunction and AD pathogenesis, suggesting that disruptions in synaptic integrity contribute to cortical thinning and cognitive decline.

Hippocampal atrophy is also a well-known feature of both normal aging and AD. Patients with AD exhibit markedly accelerated HV loss compared to age-matched older adults [89, 90], and reduced HV is closely associated with cognitive decline and progression to dementia [91–93]. Extending our primary findings on CTh, we further examined the association between *APOC1* expression and HV. We found a potential mediating role of left hippocampal atrophy in linking the *APOC1* expression and cognitive function. Notably, this effect was not observed in the right hippocampus, and no significant mediation effect was found for *APOE* expression in peripheral blood in either hemisphere. Although no statistically significant mediation effects were detected, HV marginally mediated the relationship between *APOC1* expression and cognitive outcomes (ADNI-MEM, CDRSB), whereas no such effect was observed for *APOE*. These results extend our main observations by indicating that *APOC1*’s impact on AD-related atrophy may encompass not only cortical regions but also subcortical structures that are critically involved in memory and learning processes.

*APOC1* SNPs perform similar functions as *APOE*, including roles in signal transduction, plasma lipoprotein regulation, and the transport of small molecules [35]. Specific SNPs within the *APOC1* region further differentiated conversion risk according to the presence of the variant. Among the significant variants, all except rs12691088 (i.e., rs438811, rs11568822, rs5117, rs12721046, rs12721051, rs56131196, and rs4420638) exhibited high LD with *APOE* ε4. These variants were significantly associated with conversion risk and have been reported in previous studies to exhibit strong associations with AD risk [4, 12, 77–80]. Rs12691088, the only variant independent of *APOE* ε4, has not been extensively studied. However, GWAS meta-analysis has revealed its potential association with AD risk, and it appears to be a variant concurrently implicated in both epigenetic aging and AD risk [81, 82]. These findings contribute to a deeper understanding of the impact of *APOC1* expression on AD progression and its mechanistic role in AD pathology, providing a foundation for developing new therapeutic targets. Collectively, our results suggest that *APOC1* may serve as both a potential biomarker and therapeutic target, underscoring the need for further research to elucidate its precise role in AD pathogenesis [83–88].

Although *APOE* ε4 has been identified as a key genetic risk factor for AD, it is evident that AD is a genetically heterogeneous disorder, necessitating the investigation of additional genetic contributors beyond *APOE* ε4. Our study confirmed a significant role of *APOC1* expression in accelerating CTh changes during the conversion from MCI to AD, which was further related to memory loss and cognitive decline. We examined the differential effects of *APOE* and *APOC1* on CTh changes and memory performance through spatial associations with *post-mortem* gene expression in the human brain, as well as through mediation analyses using mRNA expression levels in peripheral blood. Our findings indicated that *APOC1* mRNA expression shows a stronger association with brain atrophy and plays a more prominent mediating role in memory decline compared to *APOE*. These results align with previous research highlighting the differential impacts of these genes on cortical atrophy and memory decline, particularly in the elderly with memory impairments [25]. We speculate that *APOC1* may contribute to cortical thinning and hippocampal atrophy through molecular mechanisms that differ from those of *APOE*.

However, potential additive or synergistic interactions between *APOC1* and *APOE* cannot be ruled out. The effects previously attributed solely to *APOE* may, in part, be driven by co-expression with *APOC1*, via distinct yet converging pathways involved in lipid transport and neuroinflammation. These findings suggest that the co-expression or co-regulation of *APOC1* with *APOE* may heighten cortical vulnerability, especially in memory-related brain regions, through synergistic influences within the brain’s lipid and inflammatory environments. Multiple lines of evidence have linked *APOC1* to brain atrophy and neurodegenerative changes in AD. In a study involving elderly individuals with memory impairment, those carrying the *APOC1* risk genotype exhibited smaller HV compared to non-carriers, suggesting that *APOC1* variants may contribute to AD-like neurodegeneration [25]. Additionally, *APOC1* has been shown to exacerbate soluble Aβ oligomer-induced neuronal cell death in vitro [94], which may help explain the pronounced hippocampal and cortical atrophy observed in cognitively impaired patients. Our results reinforce these prior observations by demonstrating a significant role of *APOC1* in accelerating cortical atrophy during the conversion from MCI to AD.

Our study acknowledges the ongoing debate surrounding the roles of *APOC1* and *APOE* ε4 in AD. AD patients carrying the *APOC1* risk allele have been reported to experience faster cognitive decline and an earlier conversion from MCI to AD when they also carry the *APOE* ε4 [95]. The co-presence of *APOE* ε4 and *APOC1* risk alleles has been reported to be more strongly associated with AD than *APOE* ε4 alone, indicating that the interaction between these two genes may contribute to the development of AD and cognitive decline [96]. Conversely, other studies have shown that *APOC1* may exert an independent effect on both the risk and the pathology of AD, separate from the impact of *APOE* ε4. For example, a study found that the association of *APOC1* H2 with AD remained significant even after adjusting for *APOE* ε4 status, suggesting that the *APOC1* allele may act as an independent risk factor [15]. Additionally, a transgenic mouse study revealed that overexpression of human APOC1 protein impaired learning and memory, regardless of *APOE* expression, indicating a modulatory role for *APOC1* in AD development [94]. While several studies support a synergistic effect between *APOC1* and *APOE* ε4 in influencing AD risk, others point to *APOC1’s* independent role in AD pathology. Given these conflicting findings, additional research will be required to clarify the precise nature of the relationship between these two genetic factors.

In this study, we elucidated the role of CTh changes in the conversion of MCI to AD and highlighted *APOC1* as a potential gene that may accelerate this process. The association between *APOC1* expression and cortical thinning, particularly in regions susceptible to AD, suggests that *APOC1* contributes independently to neurodegeneration. These findings suggested that *APOC1* is a potential biomarker and therapeutic target, highlighting the need for further research on its mechanistic role in AD pathology. However, because these findings are preliminary and have not been extensively validated, further evaluation is required, such as measuring *APOC1* expression in cerebrospinal fluid (CSF) and validating the results across diverse populations and clinical settings before it can be considered for diagnostic uses. Furthermore, *APOC1’s* diagnostic performance should be directly compared with established biomarkers, such as Aβ and tau, to determine its utility. Although our findings underscore *APOC1’s* association with neurodegeneration, further studies are necessary to elucidate the mechanisms by which *APOC1* affects cortical and hippocampal atrophy, thus guiding its clinical interpretation. Prospective clinical trials investigating *APOC1* measurements for risk stratification or monitoring therapeutic response will be critical to establishing the evidence required to move toward clinical implementation.

## Limitation

Several limitations of this study must be addressed. First, the relatively small sample size limits the generalizability of our findings. Further validation in large cohorts is necessary to confirm these findings and establish *APOC1* as a biomarker across diverse populations. Second, we used only baseline gene expression measurements, which may not have fully captured the trajectory of gene expression dynamics throughout the disease progression. Third, CTh and HV were the only measures utilized to assess brain atrophy. Other neurodegenerative imaging phenotypes should be explored in future studies. Fourth, we were not able to control for systemic metabolic factors, such as cholesterol levels, when assessing the association between *APOC1* expression and CTh changes due to the limited data available in the ADNI dataset. Future studies utilizing datasets with comprehensive lipid metabolism profiles could help clarify the relationship between gene expression and neurodegeneration. Fifth, we performed a GO analysis to probe underlying biological mechanisms related to CTh changes using multiple genes significantly correlated with CTh atrophy. Although this analysis provided functional annotations that hint at processes potentially related to synaptic function, these results do not directly attribute such roles solely to *APOC1*. Future studies need to conduct mechanistic validations on *APOC1*’s impact on synaptic transmission and neuronal connectivity to unveil biological factors that may influence cortical atrophy. Sixth, *APOC1* expression was measured only at baseline, which limits the capture of potential longitudinal changes over the disease progression. Future studies incorporating repeated *APOC1* assessments are warranted to better understand its longitudinal impact on AD progression. Seventh, identifying predictive markers of AD conversion based on APOC1 expression levels and SNPs using machine learning techniques could be an interesting direction for future research. Eighth, we did not directly investigate sex-related differences in CTh changes. When we accounted for this variable in the *APOC1* adjusted regression model to mitigate potential sex-related influences, no significant association was observed with *APOC1* expression. However, future studies with larger sample sizes specifically designed to examine sex-based differences may provide deeper insight into the role of *APOC1* in AD conversion across sexes. Additionally, exploratory analyses or subgroup comparisons in subsequent research could help clarify whether sex differences meaningfully influence clinical outcomes. Lastly, we evaluated clinical potential solely based on gene expression levels in peripheral blood. However, incorporating additional molecular markers, such as expression levels in CSF, is necessary to refine future therapeutic targets and research directions. In collaboration with other institutions, ADNI is currently engaged in the multi-omics Centrally-Linked longitudinal pEripheral biomARkers of AD (CLEAR-AD) project, which will support more comprehensive analyses in future studies.

## Supplementary Information

Below is the link to the electronic supplementary material.

Supplementary file1 (DOCX 9125 KB)

## Data Availability

Imaging and phenotypic data were provided in part by the Alzheimer’s Disease Neuroimaging Initiative (https://adni.loni.usc.edu).
