# Supplementary material for: Association of APOC1 with cortical atrophy during conversion to Alzheimer’s disease
Source: GeroScience. 2025 May 15;47(6):6665–82. doi: 10.1007/s11357-025-01695-6 (PMC12638608; doi:10.1007/s11357-025-01695-6)
Supplement: Supplementary file 1 — Supplementary file1 (DOCX 9125 KB) [file 11357_2025_1695_MOESM1_ESM.docx]

**Supplementary Information**

**Table S1 | Top 100 genes associated with cortical atrophy**.

| Rank | Gene | r | p_spin_ | Rank | Gene | r | p_spin_ |
| --- | --- | --- | --- | --- | --- | --- | --- |
| 1 | *HTR2C* | -0.34126 | 0.0005 | 51 | *ELAVL1* | -0.26016 | 0.015 |
| 2 | *NR2F2* | -0.31219 | 0.009 | 52 | *EPCAM* | -0.25969 | 0.0115 |
| 3 | ***APOC1*** | -0.31093 | 0.004 | 53 | *ZNF883* | -0.25938 | 0.0125 |
| 4 | *TLL1* | -0.305 | 0.005 | 54 | *PINLYP* | -0.25903 | 0.0095 |
| 5 | *KCTD4* | -0.30135 | 0.005 | 55 | *FUOM* | -0.25822 | 0.016 |
| 6 | *CTXND1* | -0.30048 | 0.007 | 56 | *CASK* | -0.25762 | 0.009 |
| 7 | *GPD2* | -0.29833 | 0.0055 | 57 | *ARHGAP6* | -0.25695 | 0.0185 |
| 8 | *ISG15* | -0.29589 | 0.0075 | 58 | *CREG1* | -0.25563 | 0.0195 |
| 9 | *ZNF677* | -0.2936 | 0.0105 | 59 | *SESN3* | -0.25503 | 0.02 |
| 10 | *ERC2* | -0.29199 | 0.008 | 60 | *SCN9A* | -0.25461 | 0.013 |
| 11 | *SOWAHA* | -0.29102 | 0.0065 | 61 | *CACNA1E* | -0.25429 | 0.01 |
| 12 | *PALMD* | -0.29057 | 0.007 | 62 | *ARHGAP18* | -0.25422 | 0.0175 |
| 13 | *DRAIC* | -0.29053 | 0.006 | 63 | *IGSF22* | -0.2541 | 0.022 |
| 14 | *SEMA3D* | -0.29045 | 0.01 | 64 | *DNAJC12* | -0.25399 | 0.0175 |
| 15 | *CALHM6* | -0.28958 | 0.009 | 65 | *GABRE* | -0.25343 | 0.014 |
| 16 | *LRRC56* | -0.28632 | 0.008 | 66 | *TNFAIP2* | -0.25325 | 0.013 |
| 17 | *MAPK1* | -0.28585 | 0.006 | 67 | *GPR88* | -0.25286 | 0.014 |
| 18 | *LOC100129291* | -0.28522 | 0.0035 | 68 | *CPNE6* | -0.2507 | 0.018 |
| 19 | *LRRC7* | -0.285 | 0.0055 | 69 | *GLRA2* | -0.25047 | 0.018 |
| 20 | *MIR4435-2HG* | -0.28445 | 0.007 | 70 | *DIAPH2* | -0.25027 | 0.013 |
| 21 | *FNBP1L* | -0.28282 | 0.007 | 71 | *FYN* | -0.25016 | 0.0205 |
| 22 | *RGS8* | -0.28197 | 0.011 | 72 | *GRIA1* | -0.24959 | 0.0095 |
| 23 | *RAVER2* | -0.28141 | 0.012 | 73 | *CENPW* | -0.249 | 0.0175 |
| 24 | *OBSCN* | -0.28121 | 0.013 | 74 | *PTGER4* | -0.24862 | 0.0275 |
| 25 | *PPP1R1A* | -0.27642 | 0.0075 | 75 | *PDYN* | -0.24789 | 0.016 |
| 26 | *C1orf194* | -0.27602 | 0.0125 | 76 | *MLF1* | -0.24785 | 0.017 |
| 27 | *RIIAD1* | -0.27578 | 0.0115 | 77 | *TMEM200A* | -0.24784 | 0.017 |
| 28 | *IL13RA2* | -0.27505 | 0.007 | 78 | *C11orf1* | -0.24655 | 0.0155 |
| 29 | *TLE1* | -0.27393 | 0.009 | 79 | *PBX4* | -0.24596 | 0.0055 |
| 30 | *GHR* | -0.27357 | 0.01 | 80 | *PDE2A* | -0.24563 | 0.017 |
| 31 | *DDX54* | -0.27149 | 0.007 | 81 | *RALGPS2* | -0.24518 | 0.0215 |
| 32 | *SHISA7* | -0.27114 | 0.008 | 82 | *MACROD2* | -0.24516 | 0.0265 |
| 33 | *CAMK2D* | -0.26992 | 0.0185 | 83 | *DERL1* | -0.24467 | 0.018 |
| 34 | *LOC100506100* | -0.26932 | 0.015 | 84 | *HIST1H2BK* | -0.24459 | 0.026 |
| 35 | *DRD2* | -0.26873 | 0.0145 | 85 | *INPP4B* | -0.24407 | 0.0165 |
| 36 | *CAVIN3* | -0.26853 | 0.0155 | 86 | *ANK3* | -0.24377 | 0.0135 |
| 37 | *UCHL3* | -0.26805 | 0.0115 | 87 | *NSG2* | -0.24338 | 0.024 |
| 38 | *MATN2* | -0.26627 | 0.017 | 88 | *TNNT1* | -0.24328 | 0.018 |
| 39 | *CYTOR* | -0.26578 | 0.014 | 89 | *SORBS2* | -0.24323 | 0.0175 |
| 40 | *CAMKV* | -0.26529 | 0.0125 | 90 | *BFSP1* | -0.24319 | 0.0235 |
| 41 | *APBA1* | -0.26456 | 0.0165 | 91 | *PLPPR4* | -0.24295 | 0.0165 |
| 42 | *COCH* | -0.26367 | 0.01 | 92 | *GLT8D2* | -0.24269 | 0.0165 |
| 43 | *IQCJ* | -0.26331 | 0.0175 | 93 | *TMEM51* | -0.24269 | 0.0215 |
| 44 | *GNG10* | -0.26307 | 0.0135 | 94 | *ERICH6-AS1* | -0.24262 | 0.0275 |
| 45 | *NUMBL* | -0.26307 | 0.0255 | 95 | *LRRC3B* | -0.24251 | 0.027 |
| 46 | *KCNA4* | -0.26273 | 0.008 | 96 | *GSTM5* | -0.24206 | 0.0245 |
| 47 | *PLPPR5* | -0.26173 | 0.013 | 97 | *ICAM5* | -0.24198 | 0.0165 |
| 48 | *PKIB* | -0.26158 | 0.0235 | 98 | *GALNT18* | -0.24157 | 0.0245 |
| 49 | *HTR1A* | -0.26157 | 0.0125 | 99 | *HILS1* | -0.24141 | 0.02 |
| 50 | *TMOD3* | -0.2604 | 0.0135 | 100 | *PKIA* | -0.24029 | 0.029 |

**Table S2 | SNPs in *APOC1* loci and Kaplan–Meier analysis results**.

| rsID | BP | MA | HR (95% CI) | p_FDR_ |
| --- | --- | --- | --- | --- |
| rs438811 | 44,913,484 | T | 1.56 (1.05-2.30) | 0.052 |
| rs390082 | 44,913,574 | G | 0.82 (0.49-1.37) | 0.518 |
| rs11568822 | 44,914,381 | CTTCG | 1.71 (1.16-2.52) | **0.027** |
| rs12691088 | 44,915,229 | A | 3.41 (1.24-9.42) | **0.041** |
| rs5117 | 44,915,533 | C | 1.71 (1.16-2.52) | **0.027** |
| rs3826688 | 44,915,704 | T | 1.15 (0.78-1.68) | 0.518 |
| rs389261 | 44,917,086 | A | 0.27 (0.04-1.96) | 0.262 |
| rs3925681 | 44,917,843 | A | 0.75 (0.52-1.10) | 0.205 |
| rs12721046 | 44,917,997 | A | 1.65 (1.13-2.42) | **0.028** |
| rs12721056 | 44,918,487 | T | 0.75 (0.52-1.10) | 0.205 |
| rs484195 | 44,918,620 | A | 1.09 (0.74-1.60) | 0.659 |
| rs12721052 | 44,918,715 | A | 0.75 (0.52-1.10) | 0.205 |
| rs12721051 | 44,918,903 | G | 1.65 (1.12-2.41) | **0.028** |
| rs1064725 | 44,919,304 | G | 0.76 (0.35-1.63) | 0.518 |
| rs56131196 | 44,919,589 | A | 1.71 (1.16-2.49) | **0.027** |
| rs4420638 | 44,919,689 | G | 1.71 (1.16-2.49) | **0.027** |

Abbreviations: SNP, single-nucleotide polymorphism; BP, base-pair location in hg38 coordinates; MA, minor allele of variant; HR, hazard ratio; CI, Confidence Interval

**
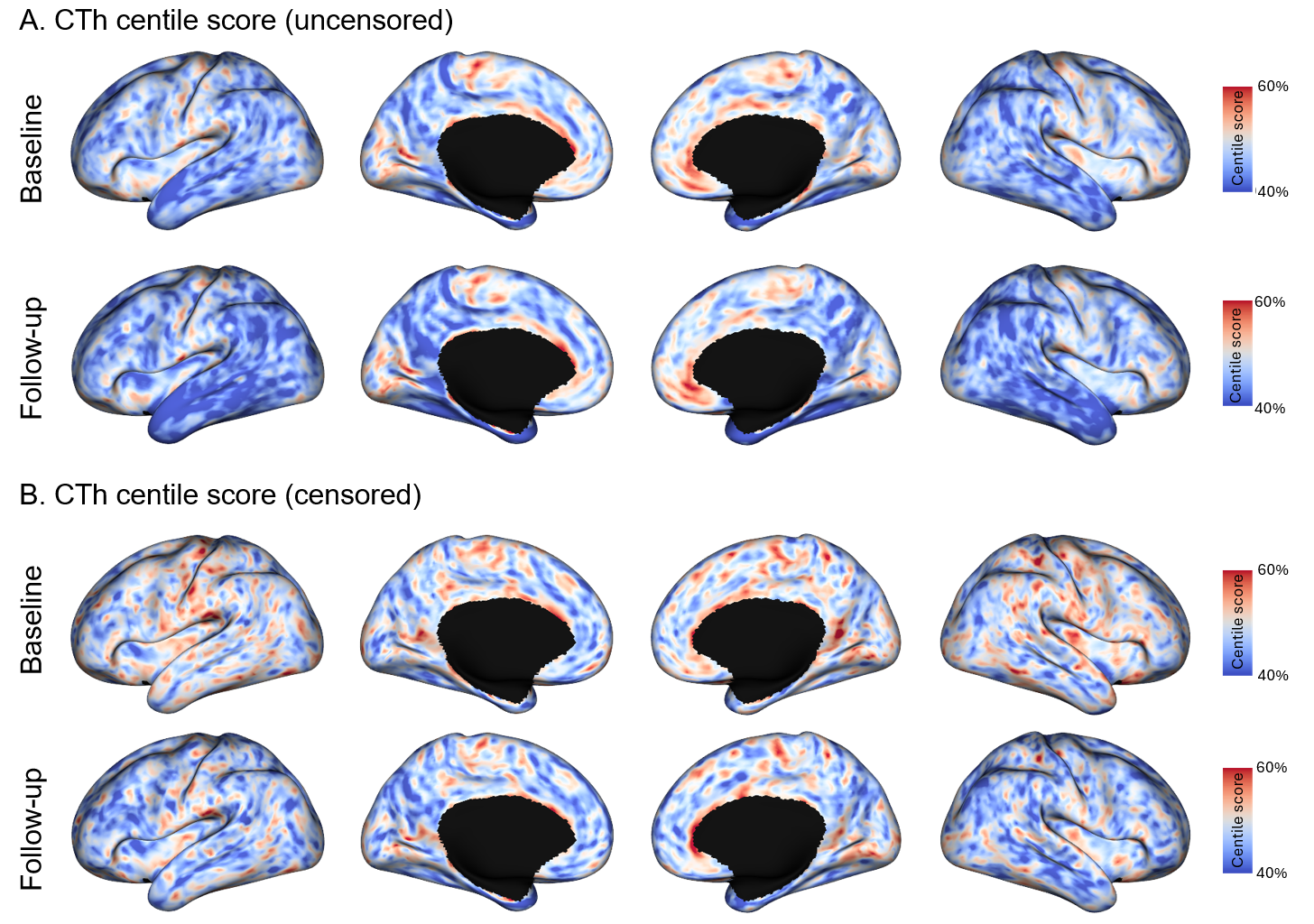
**

**Fig. S1 | CTh centile score for baseline and follow-up for two subgroups. (A)** CTh centile score of MCI with conversion to AD (uncensored) and **(B)** stable MCI (censored). The scores at baseline and follow-up are displayed on brain surfaces.
Abbreviation: CTh, Cortical thickness**;** MCI, mild cognitive impairment; AD, Alzheimer’s disease.


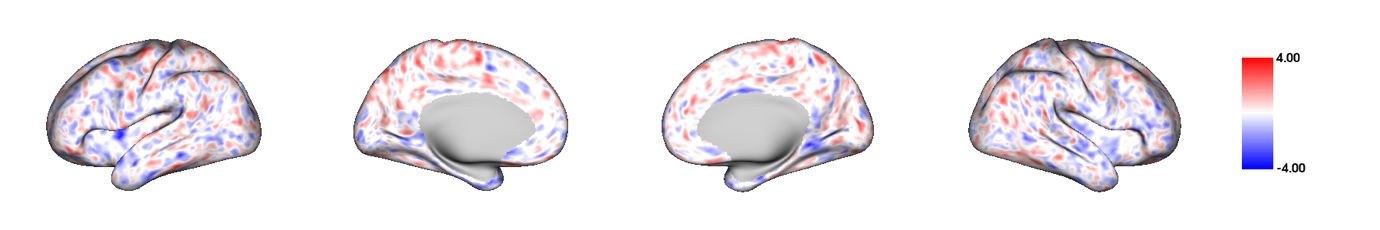


**Fig. S2 | Comparison of baseline CTh centile scores between the low and high *APOC1* expression groups.** T-statistics are displayed on brain surfaces, and no regions survived after the multiple comparison correction.


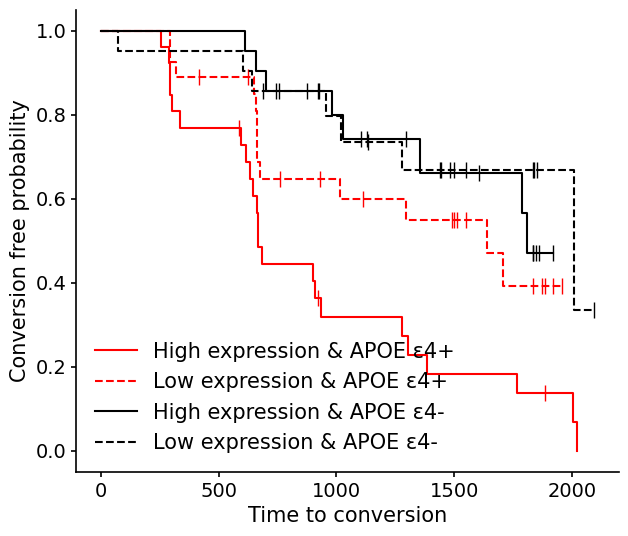


**Fig. S3 | Time to conversion analysis using groups based on *APOC1* expression levels and the APOE ε4 allele status.** Kaplan-Meier plot is displayed for the four groups stratified based on APOC1 expression levels (high or low) and the APOE ε4 allele status (positive or negative).


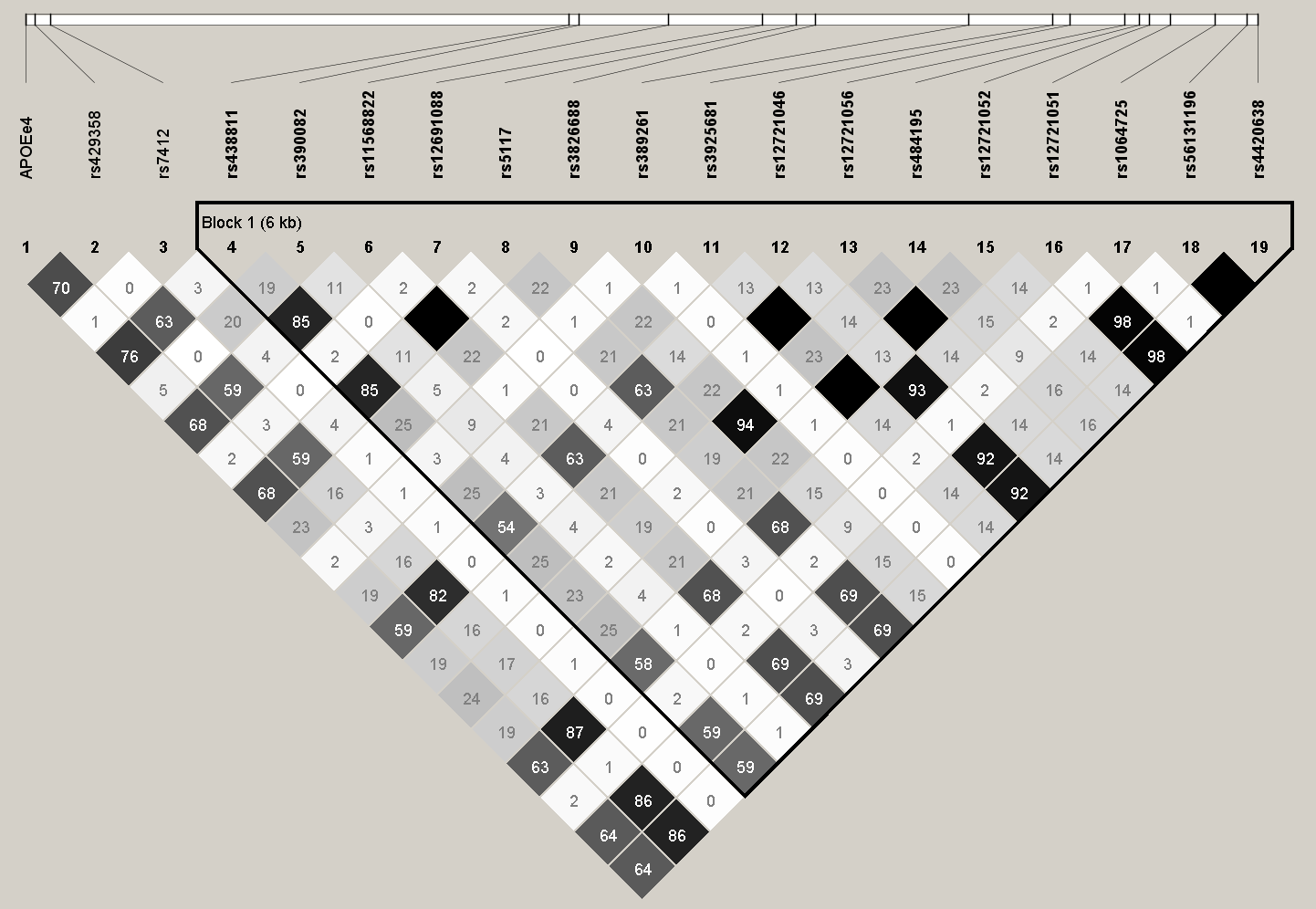


**Fig. S4 | Linkage disequilibrium pattern between *APOE* ε4 and *APOC1* locus.** Darker colors indicate stronger linkage disequilibrium relationships, with each value representing $r^{2}$. Among the loci, rs429358 and rs7412 constitute components of *APOE* ε4, while the remaining loci represent *APOC1* SNPs.

**
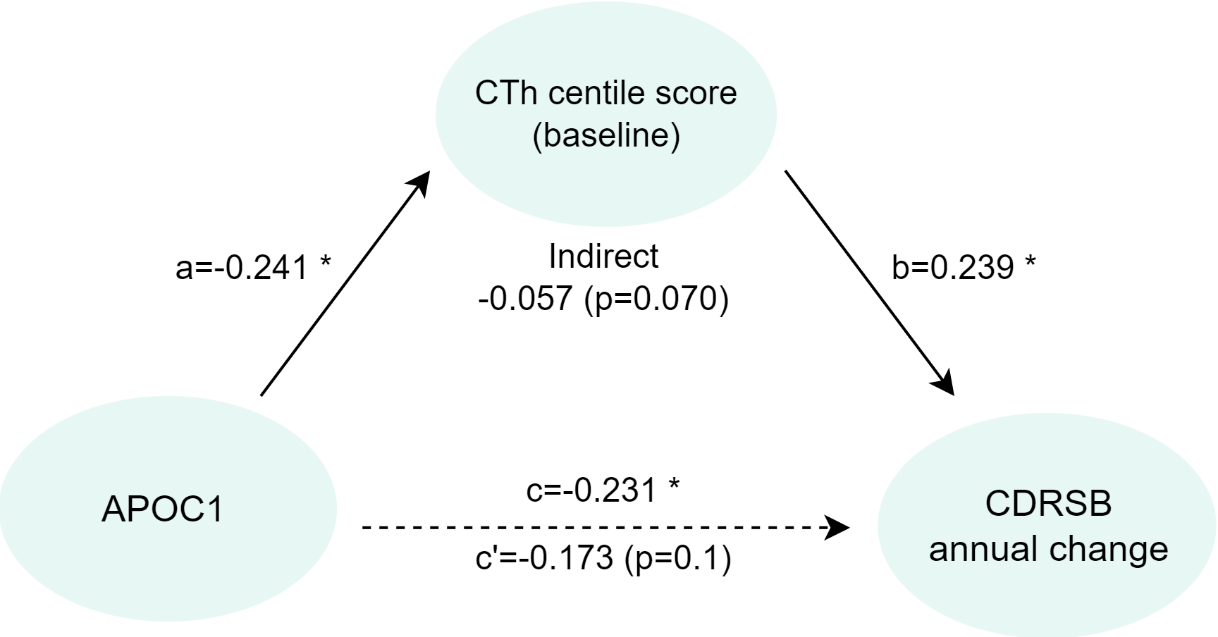
**

**Fig. S5 | Mediation analysis using CDRSB.** Mediation analysis assessed the direct and indirect effects of the *APOC1* expression on the annual change of CDRSB, mediated by the CTh centile score. Reported values are regression weights with significance in asterisks. * denotes a p-value <0.05.
Abbreviation: CDRSB, clinical dementia rating scale sum of boxes; CTh, Cortical thickness.


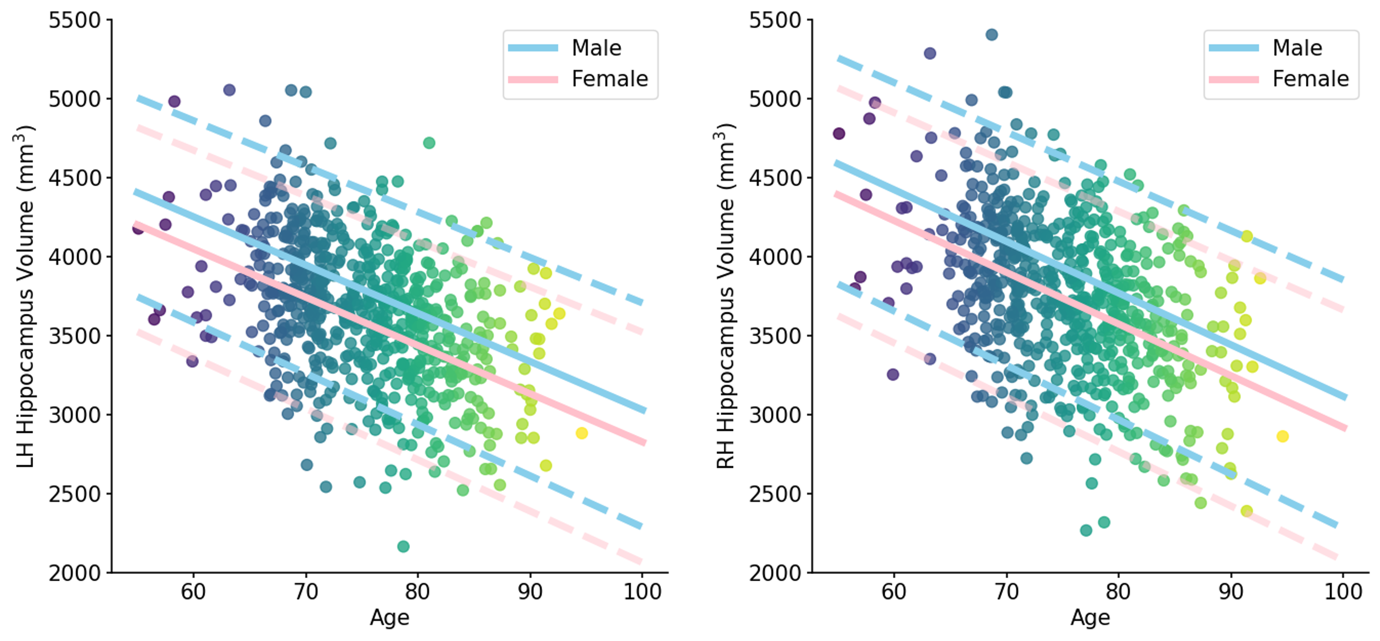


**Fig. S6 | Hippocampal volume changes across ages**. Hippocampal volume of each hemisphere is plotted across ages in 636 individuals with CN status. The colored dot indicates the age of the participants with CN status, and sky blue and pink lines represent normative curves of males and females, respectively.
Abbreviations: LH, left hemisphere. RH, right hemisphere.


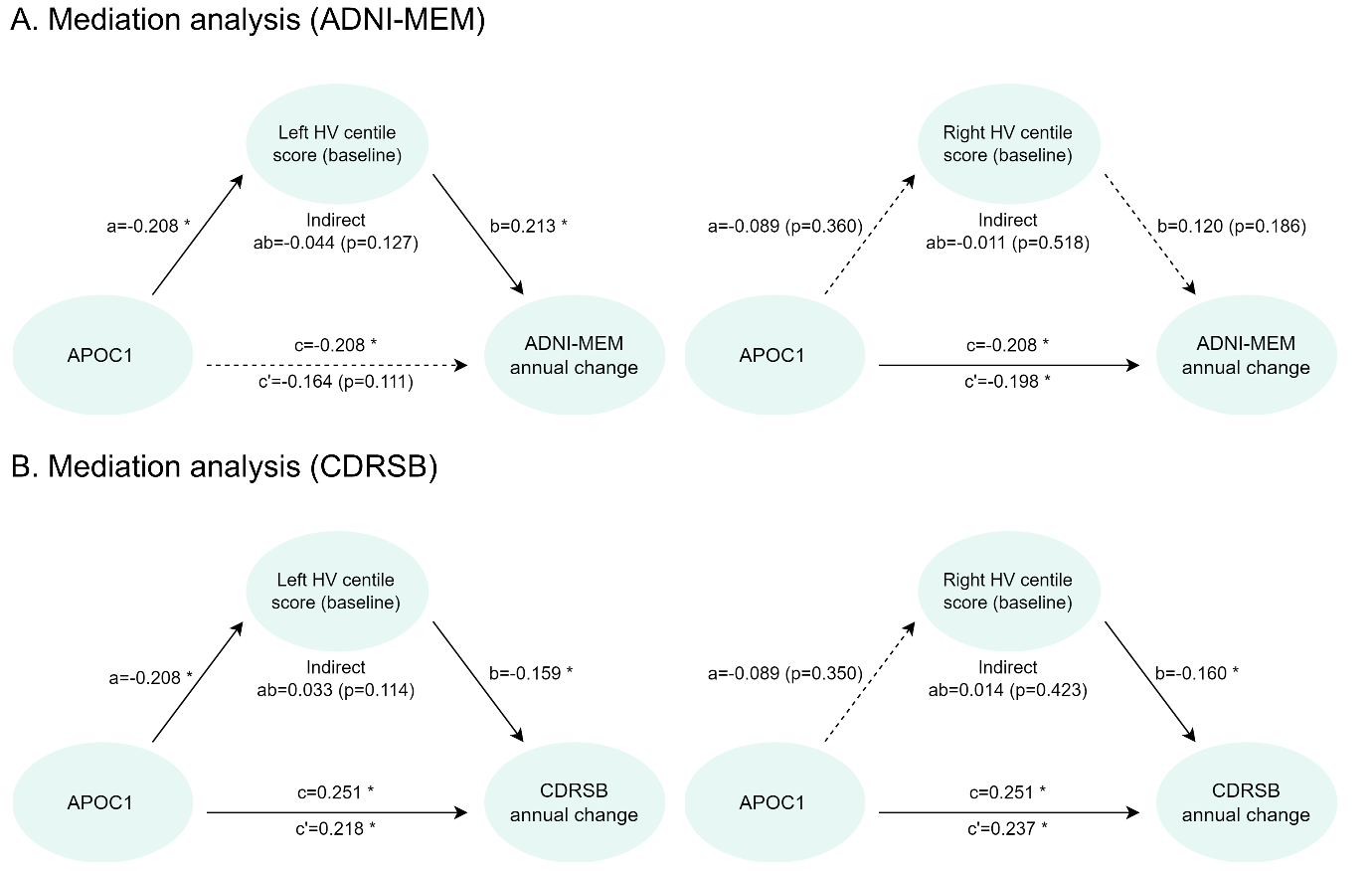


**Fig. S7 | Mediation analysis using hippocampal volume for *APOC1*.** Mediation analysis assessed the direct and indirect effects of the APOC1 expression on the annual change of **(A)** ADNI-MEM and **(B)** CDRSB, mediated by the left and right hippocampal volume centile score. Reported values are regression weights with significance in asterisks. * denotes a p-value <0.05.
Abbreviations: CDRSB, clinical dementia rating scale sum of boxes; CTh, Cortical thickness; HV, Hippocampus volume.

**
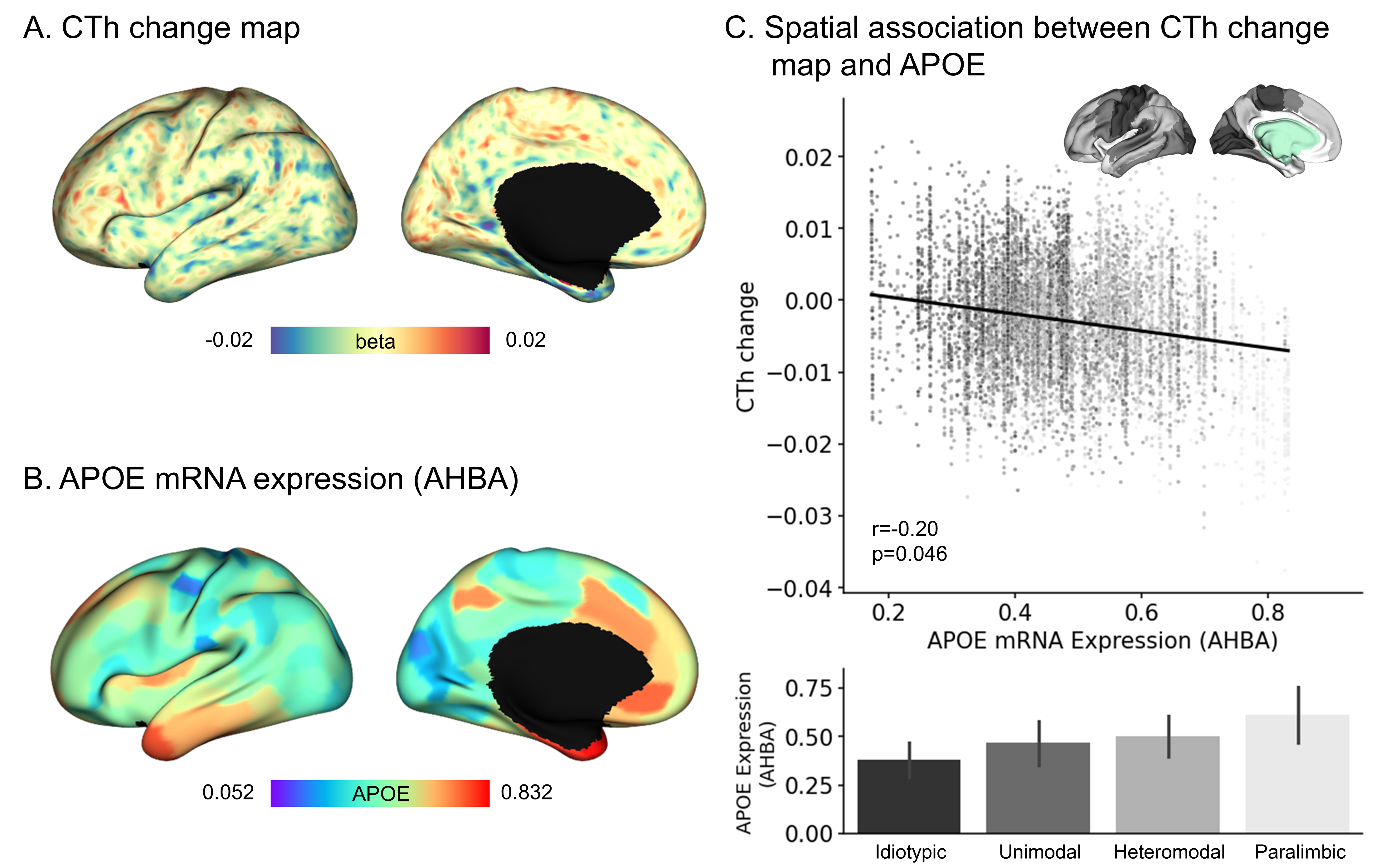
**

**Fig. S8 | Spatial correlation between CTh change and *APOE* expression. (A)** CTh change map and **(B)** *APOE* mRNA expression are demonstrated on brain surfaces. **(C)** A **s**patial correlation between the CTh change map and *APOC1* expression is illustrated with a scatter plot. The color was coded according to prior models of cortical hierarchy. The bar plot indicates the *APOE* expression according to cortical hierarchies.

Abbreviation: CTh, Cortical thickness; AHBA, Allen Human Brain Atlas.

**
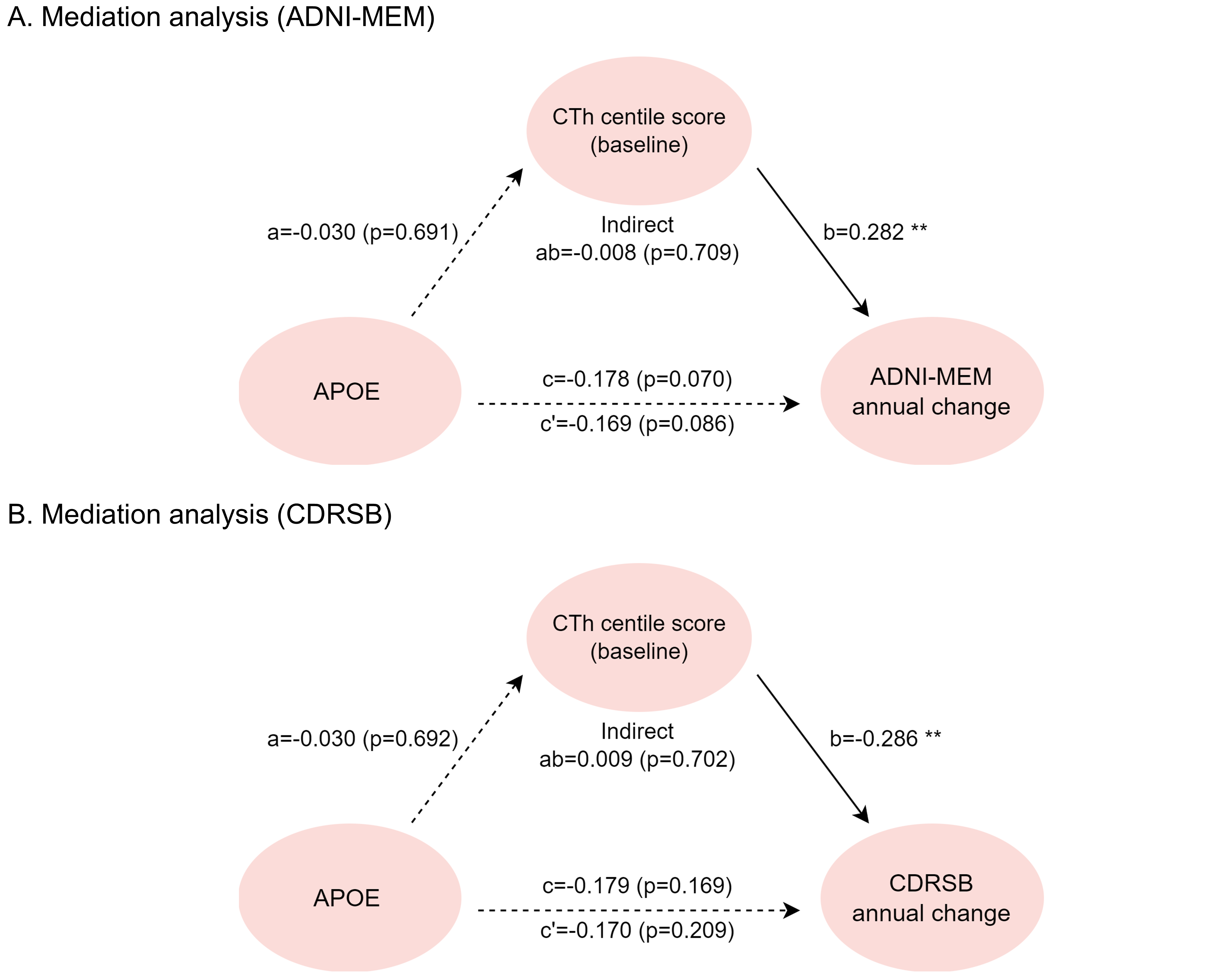
**

**Fig. S9 | Mediation analysis using *APOE*.** Mediation analysis assessed the direct and indirect effects of the *APOE* expression on the annual change of ADNI-MEM and CDRSB, mediated by the CTh centile score. Reported values are regression weights with significance in asterisks. * denotes a p-value <0.05.
Abbreviations: CTh, Cortical thickness; CDRSB, clinical dementia rating scale sum of boxes; ADNI-MEM, Alzheimer’s disease Neuroimaging Initiative composite MEMory score.


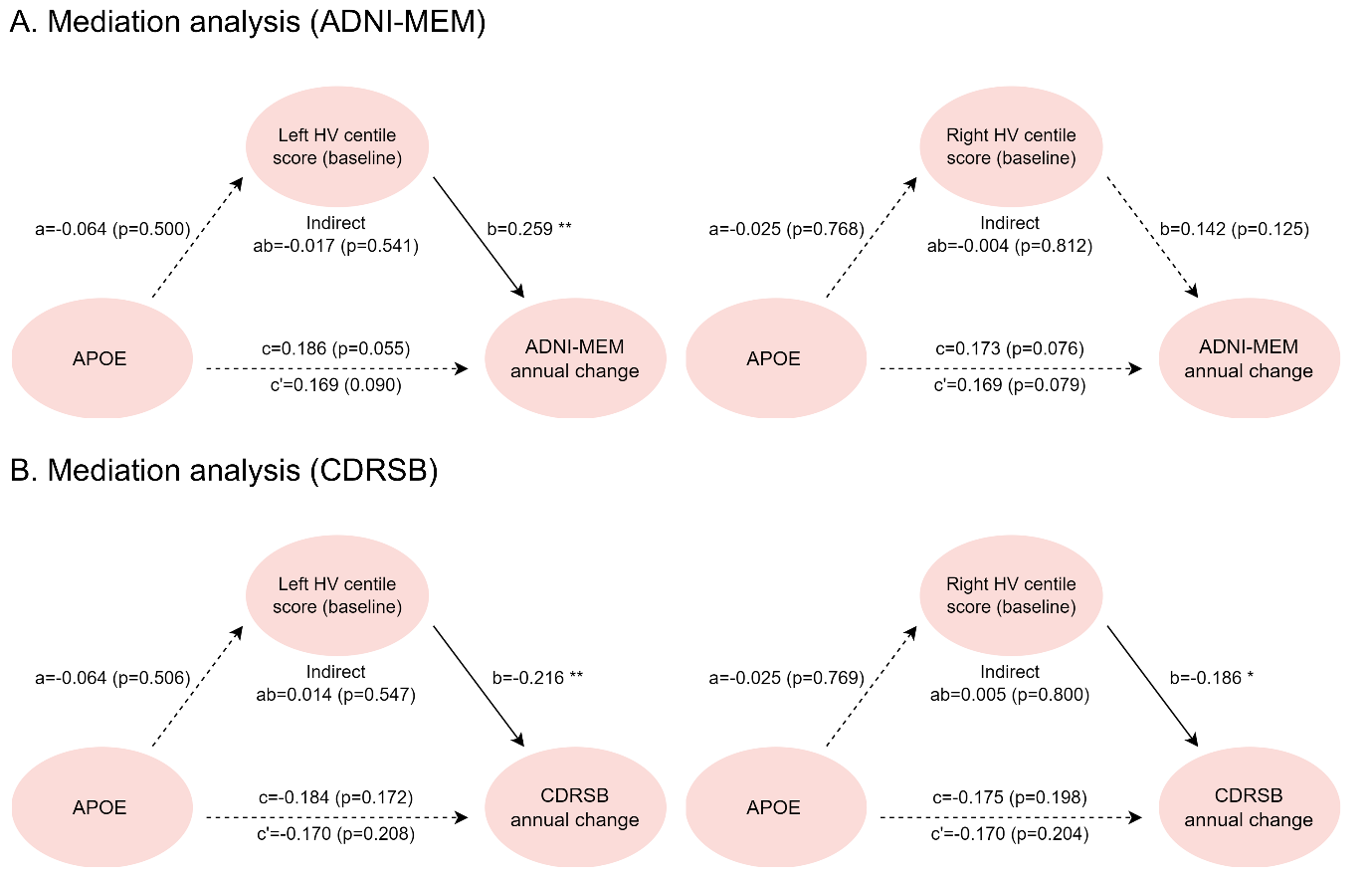


**Fig. S10 | Mediation analysis using hippocampal volume for *APOE*.** Mediation analysis assessed the direct and indirect effects of the APOE expression on the annual change of **(A)** ADNI-MEM and **(B)** CDRSB, mediated by the left and right hippocampal volume centile score. Reported values are regression weights with significance in asterisks. * denotes a p-value <0.05.
Abbreviations: CDRSB, clinical dementia rating scale sum of boxes; CTh, Cortical thickness; HV, Hippocampus volume.
